# Pattern and extent of off-label and unlicensed drug use in neonatal intensive care units in Iran

**DOI:** 10.1186/s12887-018-1370-x

**Published:** 2019-01-04

**Authors:** Leila Kouti, Maryam Aletayeb, Seyyed Mohammad Hassan Aletayeb, Amir Kamal Hardani, Kaveh Eslami

**Affiliations:** 1 Department of Clinical Pharmacy, Ahvaz Jundishapur University of Medical Sciences, Ahvaz, Iran; 2 Student Research Committee, Ahvaz Jundishapur University of Medical Sciences, Ahvaz, Iran; 3 Department of Pediatrics, Faculty of Medicine, Imam Khomeini Hospital, Ahvaz Jundishapur University of Medical Sciences, Ahvaz, Iran; 4 Department of Pediatrics, Faculty of Medicine, Abuzar Children’s Hospital, Ahvaz Jundishapur University of Medical Sciences, Ahvaz, Iran

**Keywords:** Newborn, NICU, Off-label, Unlicensed, Drug use

## Abstract

**Background:**

Many newborns may need to be hospitalized and receive drugs during the first days of their lives. These drugs are fundamentally prescribed as off-label and unlicensed. This study aimed to investigate the amount of these kinds of drugs administered in the Neonatal Intensive Care Units (NICUs) of Abuzar and Imam Khomeini Teaching Hospitals in Ahvaz, Iran.

**Methods:**

This was a 3-month descriptive, cross-sectional study with retrospective nature in which 193 hospitalized newborns were studied. Demographic data were extracted from the patients’ files. The drugs were classified as off-label, unlicensed or licensed according to the Pediatric & Neonatal Dosage Handbook (Lexicomp®, 22nd Edition).

**Results:**

In total, 1049 prescriptions were registered for the 193 hospitalized newborns (term and preterm). For each newborn, the mean numbers of prescriptions and drugs received were 5.4 and 4.5, respectively. The mean numbers of prescriptions and drugs were greater for preterm newborns.

Of the total 1049 prescriptions, 38.1% were off-label and 1.9% were unlicensed. Of the 193 newborns, 85% received at least one off-label or unlicensed prescription. Off-label prescriptions were mostly related to dose (44.8%) and dosing interval (36.5%). Most off-label drugs were antibiotics (mainly Gentamicin). Albuterol was used off-label in 100% of the cases.

**Conclusions:**

The results of the present study show that the prescription of off-label and unlicensed drugs in NICUs is as high in Iran as in other countries. This suggests that it is necessary to provide information to neonatologists to decrease the prescription of off-label and unlicensed drugs.

## Background

In the newborn period, which is generally defined as the first 28 days of life, hospitalized newborns need to receive multiple medications to survive. Many of these medications can be used in older children and adults, but due to the unique physiology of neonates, the pharmacokinetic findings of drugs in older patients cannot be generalized to this group. Therefore, the appropriate dose of many drugs administered to neonates is unknown. Neonates are exposed to non-standard formulations, errors in drug dose adjustments and adverse drug reactions. Other risks for neonates include the higher risk of death or the emergence of serious complications throughout life due to the administration of these medicines [1–3].

The risk factors of adverse drug reactions (ADRs) in neonates are poorly understood. One of the concerns associated with this issue is the use of off-label and unlicensed drugs in this age group [4].

Off-label use refers to drugs administered outside their marketing authorization (product license) in terms of age, dose, dosing interval, route of administration or indication. Unlicensed use refers to the modified administration of licensed drugs. This can include changes in the formulation due to the lack of a suitable formulation for newborns, such as the use of Furosemide tablets in the form of an oral solution [5–7]. The prescription of off-label drugs is not unlawful nor necessarily incorrect and exists in some neonatal care protocols. However, there may be dangers such as ineffectiveness, complications or even death. Evidence suggests an increased risk of unwanted side-effects associated with the use of off-label drugs [8]. Therefore, the decision to prescribe these drugs should be evaluated according to available clinical treatments, therapeutic options, and profit-and-loss analysis [9].

Many neonatologists currently prescribe off-label drugs routinely, although most of them believe that information about the risks and benefits of using off-label drugs in neonates is inadequate. According to various studies conducted in NICUs, the lack of knowledge available to physicians has led to the extensive prescription of off-label and unlicensed drugs [5, 10–12].

There are contrasting results regarding the prevalence of using off-label and unlicensed drugs in neonates. In some previous studies, the rate of using these drugs has been reported to be 55-80% [13–15]. This variation in the prevalence is due to a variety of factors, including differences in the designs and methods of the studies and the various definitions of off-label and unlicensed drugs in these studies [4].

Given the need to reduce the prescription of off-label and unlicensed drugs in NICUs revealed by previous studies, and the available information indicating a lack of such studies in Iran, we aimed to evaluate the extent of off-label and unlicensed drugs prescribed to neonates admitted to the NICUs of two Teaching Hospitals in Ahvaz, Iran and to compare the results between preterm and term neonates.

### Ethics approval

Entry into the study was initially approved by the Ethics Committee of the Research Deputy of Jundishapur University of Medical Sciences in Ahvaz according to the code of ethics: IR.AJUMS.REC.13494.531.

## Methods

### Description and design of the study

This was a retrospective, descriptive, cross-sectional study in which the drugs prescribed to neonates admitted to the level 3 NICUs of Abuzar Children’s Teaching Hospital and Imam Khomeini Teaching Hospital in Ahvaz, southwest Iran were examined during a three-month period from 1 January to 31 March 2016.

The inclusion criteria of the study were as follows: Neonates younger than 28 days old who had been admitted to the NICU for at least 24 h and had received at least one medication to prevent or treat a specific disease. Moreover, their complete clinical record had to be available during the study. Patients with incomplete records, as well as neonates whose medical record had indicated only the use of oxygen therapy, vaccines, blood products (except immunoglobulin), vitamins, electrolytes, total parenteral nutrition, and intravenous hydration were excluded. No intervention was performed on patients. Entry into the study was initially approved by the Research Ethics Committee of Ahvaz Jundishapur University of Medical Sciences (AJUMS) according to the code of ethics: IR.AJUMS.REC.13494.531.

### Data collection

The patients’ demographic data, including gestational age (GA), route of delivery, birth weight, gender, age at admission, number of hospitalization days, diagnoses, types of treatment and types of discharge (recovery, death), were determined by studying the patients’ hospital records.

The drug information, including the names of drugs, the number of drugs, the drug categories, the dosage forms, the doses, the dosing intervals, the routes of administration, the indications, the duration and the frequency of administration of the drugs, were extracted from the records of the hospitalized patients and transferred to the specified forms. The data were collected by the researcher. As we did not record personal identifying data for the neonates and no intervention was performed on patients, we did not seek consent for participation in this study.

In this study, neonates were divided into two groups, based on their GA:

- Preterm neonates: < 37 weeks GA, which itself consisted of 2 subgroups:
  1- 1 <  32 weeks GA
  1- 2 32-36 weeks GA
- Term neonates: ≥37 weeks GA

Moreover, neonates were divided into three groups based on their birth weight:

1. Very low birth weight (< 1500 g)
2. Low birth weight (< 2500 g)
3. Normal birth weight (≥2500 g)

### Classification of drugs

All drugs prescribed to patients were reviewed for inclusion in one of these categories: off-label, unlicensed, licensed.

Previous studies with the same purpose have used different definitions for off-label and unlicensed drugs.

In the current study, off-label use was defined as *the administration of a drug in a different manner from those recommended in the marketing authorization in terms of age, dose, dosing interval, route of administration, or indication*.

An unlicensed drug was defined as *a drug without a product license, a modified form of a licensed drug, which was administered after a change in the formulation* [5–7].

The third category included licensed drugs, which were prescribed and administered, following the terms of the marketing authorization.

Drugs that were prescribed as off-label for only part of the treatment period were classified as off-label drugs. The categorization of drugs in terms of the type of prescription (off-label, unlicensed, licensed), was done based on the gestational age, the birth weight, and the post-natal age of the neonates, according to the source of prescription, The Lexicomp Pediatric & Neonatal Dosage Handbook, 22nd edition [16], which is a universal resource for clinicians treating pediatric and neonatal patients.

We studied the availability of drug information in the handbook for each prescription. If no information was available with regard to administration of the drug in neonates and/or preterm neonates, or the drug was approved for administration in a different age group; we categorized the drug as off-label for age. If a drug was considered off-label for age, it was not further assessed for other off-label categories. If the drug information was available, but the administration was recommended for a higher or lower dose, we defined the drug as off-label for dose. In the cases that the drug information suggested the administration of the drug for another dosing interval, route of administration or indication, the drug was classified as off-label in terms of dosing interval, route of administration or indication.

### Statistical analysis of data

Data were recorded in Microsoft Office Excel, and the statistical analysis was performed with SPSS V.20. To describe the data, the mean and standard deviation (SD) were used for quantitative variables, and frequency and percentage were used for qualitative variables. Data were analyzed by t-test and variance analysis (in the case of the assumption of normality), and Mann-Whitney U test, Kruskal-Wallis test, chi-square test or Fisher’s exact test (in the case of the absence of normality). A *p*-value less than 0.05 indicated statistical significance.

## Results

### Population

During the study, the hospital files of 193 neonates who were hospitalized for at least 24 h and had complete clinical records were evaluated. Of the total number of neonates, 59.1% were male, 53.9% were preterm and 62.2% were outborn. Regarding the type of delivery, 64.2% of neonates were born via cesarean section (C/S).

The mean gestational age of the neonates was 34 ± 4.4 weeks (ranging from 24 to 40 weeks), and the mean gestational age of the preterm neonates was 31 ± 3.2 weeks. The mean number of hospitalization days was 10.6 ± 9.8 (ranging from 1 to 69 days), with a total of 2040 days of hospitalization for all the neonates (Table 1).

**Table 1:** Demographic and clinical data of neonates admitted to the NICUs

Demographics	Preterm Neonates		Term Neonates	Total Neonates	*P* value
	< 32 weeks GA	32-36 weeks GA	≥37 weeks GA	–	
Neonates admitted, No	47	57	89	193	–
Gender, No. (%)					–
Male	26 (25)	35 (33.6)	53 (59.6)	114 (59.1)	
Female	21 (20.2)	22 (21.2)	36 (40.4)	79 (40.9)	
Place of birth, No. (%)					< 0.001*
Inborn	43 (41.3)	28 (27)	2 (2.2)	73 (37.8)	
Outborn	4 (3.8)	29 (27.9)	87 (97.8)	120 (62.2)	
Route of delivery, No. (%)					0.002*
Vaginal	11 (10.6)	16 (15.4)	42 (47.2)	69 (35.8)	
C/S	36 (34.6)	41 (39.4)	47 (52.8)	124 (64.2)	
Birth weight(g), Mean ± (SD)	1201(±372.6)	2169(±405.5)	3317(±405.5)	2463(±954.9)	< 0.001*
Hospitalization days, Mean ± (SD)	22.2(±18.1)	8.4(±5)	5.8(±4.3)	10.6(±9.8)	< 0.001*
Discharged, No. (%)					0.001*
Recovery	33 (31.7)	51 (49)	86 (96.6)	170 (88.1)	
Death	14 (13.5)	6 (5.8)	3 (3.4)	23 (11.9)	
Post-natal age (days), Mean ± (SD)	1.9 ± 0.4	6.8 ± 4.1	14 ± 10.1	10 ± 8	< 0.001*

*SD* Standard deviation; *, *p*-value < 0.05

The most common diagnoses for neonates were sepsis (34.2%), respiratory distress syndrome (RDS) (23.3%), transient tachypnea of newborns (TTN) (11.9%), pneumonia (9.8%), and bronchiolitis (5.9%).

We classified all prescriptions in the following therapeutic groups, in descending order of frequency: respiratory (39.9%), infectious (31.1%), gastrointestinal (8.8%), neurological (8.3%), metabolic (4.1%), cardiovascular (3.1%), nephrologic (2.1%), hematologic (1.5%), and others (1.1%).

### Prescription of medications

A total of 1049 prescriptions, including 72 different formulations and 59 drugs, were registered for newborns. For each neonate, the mean number of prescriptions received was 5.4 ± 4.1 (minimum = 1, maximum = 24), and the mean number of drugs needed was 4.5 ± 3 (minimum = 1, maximum = 17). The number of prescriptions for each neonate was higher for preterm neonates than for term neonates (*p*-value = 0.003). Furthermore, preterm neonates received more drugs than term neonates (*p*-value < 0.001) (Table 2).

**Table 2:** Characteristics of prescriptions for neonates in the NICUs

Characteristics of prescriptions	Preterm Neonates		Term Neonates	Total Neonates	*P* value
	< 32 weeks GA	32-36 weeks GA	≥37 weeks GA	–	
Prescriptions,No. Mean (±SD)	8 (±4.8)	4.8 (±3.8)	4.5(±3.3)	5.4 (±4.1)	0.003*
Drugs,NoMean (±SD)	6.6 (±3.4)	4 (±2.7)	3.6 (±2.3)	4.5 (±3)	< 0.001*
Off-label prescriptions, No. (%)	96 (25.7)	115 (42)	188 (46.9)	399 (38.1)	* < 0.001
Unlicensed prescriptions, No. (%)	1 (0.3)	7 (2.6)	12 (3)	20 (1.9)	*0.043
Neonates with off-label prescription, No. (%)	41 (87.2)	48 (84.5)	75 (84.3)	164 (85)	0.8
Neonates with unlicensed prescription, No. (%)	1 (2.1)	7 (12.3)	9 (10.1)	17 (8.8)	0.554
Neonates with off-label or unlicensed prescription, No. (%)	41 (87.2)	48 (84.5)	75 (84.3)	164 (85)	0.8

*SD* Standard deviation; *, *p*-value < 0.05

In our study, out of the 1049 prescriptions registered, 38.1% were off-label and 1.9% were unlicensed, which were received by 164 (85%) and 17 (8.8%) of the newborns, respectively. Of the 193 newborns, 85% received at least one off-label or unlicensed prescription. 60% of the prescriptions were licensed, all of which were prescribed in accordance with our reference standards.

According to Table 2, patients received more off-label drugs than unlicensed ones, and the frequency of prescribing off-label drugs was significantly higher than that of prescribing unlicensed drugs.

Most prescriptions classified as off-label were in terms of dose and dosing interval, respectively, with 44.8% of the off-label prescriptions regarding the dose and 36.5% regarding the dosing interval. Other off-label prescriptions, based on their frequency, were in terms of indication (11.9%), age group (5.3%), and route of administration (1.5%).

The most widely prescribed off-label drugs in NICUs, mainly antibiotics, are listed in Table 3. Many of these drugs were off-label for more than one reason (Table 3). Albuterol and Gentamicin were the drugs most commonly prescribed as off-label in term and preterm neonates, respectively. In terms of dose and dosing interval, Albuterol was prescribed off-label in 100% of cases.

**Table 3:** The ten most frequently prescribed off-label or unlicensed drugs

Drug	Number (%) of total prescriptions	Reason(s) for off-label/unlicensed status
Ampicillin	174 (16.6)	Off-label: Dose/Dosing Interval
Gentamicin	102 (9.7)	Off-label: Dose/Dosing Interval
Caffeine	82 (7.8)	Off-label: Age Group
Albuterol	73 (7)	Off-label: Dose/Dosing Interval/Indication
Cefotaxime	71 (6.8)	Off-label: Dose/Dosing Interval/Indication
Vancomycin	48 (4.6)	Off-label: Dose/Dosing Interval
Furosemide	43 (4.1)	Off-label: Dose/Indication Unlicensed: Modified Formulation
Phenobarbital	39 (3.7)	Off-label: Dose/Dosing Interval/Indication Unlicensed: Modified Formulation
Ranitidine	25 (2.4)	Off-label: Dose/Indication
Amikacin	19 (1.8)	Off-label: Dose/Dosing Interval/Indication

Table 4 shows the status of off-label and unlicensed prescriptions in neonates with different birth weights.

**Table 4:** Descriptionof off-label and unlicensed prescriptions in neonates according to their birth weight

Characteristics of prescriptions	Birth Weight			*P* value
	< 1500 g	< 2500 g	≥2500 g	–
Off-label prescription, No. (%)	29 (82.9)	48 (88.9)	87 (83.7)	0.634
Unlicensed prescription, No (%)	2 (5.7)	5 (9.3)	10 (9.6)	0.773
Neonates with off-label or unlicensed prescription, No (%)	29 (82.9)	48 (88.9)	87 (83.7)	0.634

All unlicensed drugs were orally administered in our study. Phenobarbital tablet that was dissolved, after changing in the formulation, was the most common unlicensed drug prescribed to neonates.

In our study the most common routes of administration included intravenous injection (72.7%), oral administration (11.2%), inhalation (7.8%) topical (2.3%), intratracheal injection (2.2%), ocular (2%), subcutaneous (0.8%), rectal (0.7%), and nasal (0.3%).

## Discussion

To the best of our knowledge, this study was the first to investigate the extent of off-label and unlicensed drugs prescribed in Neonatal Intensive Care Units (NICUs) in Iran. Our study was conducted in two NICUs, one with neonates referred from other centers (outborn) and the other with neonates born in the same hospital (inborn).

The mean gestational age of the neonates in our study was 34 weeks, which is similar to the results reported previously in Germany, Ireland and France [3, 16, 17]. The rate of cesarean delivery of the preterm neonates was higher in our study than that of the term neonates. (*P*-value = 0.002), which is similar to the results of a previous study in Portugal [18]. The higher prevalence of cesarean delivery in high risk preterm neonates is clearly to prevent severe vaginal delivery complications.

In this study, the mean hospitalization length for neonates in the NICU was 10.6 days, which was the same as in a study by Jana Lass et al. in Estonia (10 days) and less than in some other studies [3, 17–19]. The most common cause of hospitalization for neonates in this study was sepsis, while in two similar studies, the most common causes of the hospitalization were neonatal hyperbilirubinemia and respiratory distress [19, 20].

In the present study, as in some previous studies, according to the Anatomical Therapeutic Chemical (ATC) Classification System, systemic antibiotics were the most commonly prescribed drugs (*n* = 463, 44.1%) [3, 21].

In our study, out of the total 1049 prescriptions, 38.1% were off-label, which is similar to the results of studies in the Slovak Republic (43%) [22], Spain (41.4%) [23] and Finland (36%) [5]. It is a lower percentage than was found in other studies conducted in France (59.5%) [17], Portugal (52.7%) [19] and Australia (47%) [20]; however, it is a higher percentage than was identified in a study performed in Switzerland (25%) [24].

The unlicensed prescriptions in this study were 1.9% of the total prescriptions, which was significantly lower than the prevalence identified in most of the previous studies [5, 22–24].

This variation in results could be due to the application of different methods in this study and different definitions of off-label and unlicensed drugs used in other studies.

This study found that 85% of neonates admitted to the NICU received at least one off-label or unlicensed drug, which was a similar result to that of a study done in a NICU in Australia (80%) [20] and close to the results of some similar studies conducted in recent years (69.7-100%) [5, 17, 18, 24]. Ampicillin and Gentamicin were the most commonly prescribed off-label drugs in the present study, which are the same drugs identified in a study in Spain [23]. This finding was different from that of a similar study previously performed by Salehifar et al. in Iran with the purpose of determination of antibiotics consumption, in which the most and the least frequently used antibiotics were ceftriaxone and gentamicin, respectively [25].

The high prevalence of prescribing off-label drugs is not only due to the lack of clinical evidence, but also to the lack of complete information in neonatal references. We showed in this study that the prescription of off-label and unlicensed drugs was significantly higher in term neonates than in preterm neonates (46.9% vs. 32.6%); this finding is similar to those of two studies conducted in recent years [19, 21]. However, among preterm neonates, off-label dugs were given to a higher percentage of newborns with gestational age of less than 32 weeks (87.2%) than to neonates with gestational age of 32-36 weeks (84.5%). In general, similar percentages of term and preterm neonates were exposed to off-label or unlicensed drugs, which indicated that in the present study, the gestational age did not significantly affect whether the neonates received this type of drugs. However, with regard to drug exposure, gestational age had a significant effect, and preterm neonates received more drugs than term neonates.

In this study the most common off-label drug prescribed was related to dose, which was similar to the findings of a study from Portugal, [18] and different from the findings of studies from Australia, Spain and Norway, in which the most common off-label prescription of drugs were related to indication, age and route of administration [20, 23, 26]. Because of the lack of suitable oral forms of drugs for neonates, splitting tablets and dissolving them in sterile fluids was the most common form of unlicensed prescription in our study. Dissolved Phenobarbital was the most commonly prescribed unlicensed drug in this study.

In the outborn group of neonates at the NICU, given the winter season and the epidemic of respiratory diseases (bronchiolitis), many neonates older than 7 days presented with respiratory distress symptoms, and Albuterol inhalation was repeatedly administered, which were prescribed off-label in 100% of cases, both in terms of dose and dosing interval. However, it seems that its administration was unnecessary for many neonates.

The frequent and long-term administration of antibiotics was found in both NICUs, which is unsurprising, because the most common diagnoses were sepsis, RDS and TTN. This use of antibiotics requires review and limitation according to in-hospital or national guidelines. The use of Bactec to perform cultures and to obtain their results in the shortest possible time should be considered, as well as the discontinuation of antibiotics in the event of negative cultures and the absence of risk factors.

This study showed that the prevalence of prescription of off-label and unlicensed drugs in our country is similar to that of reported in many other countries. The special conditions of neonates, especially preterm neonates, may be the main cause of these kind of prescriptions. To avoid this issue and to support the appropriate drug therapy in a high-risk group such as newborns, the first step is to provide relevant information about the risks and unwanted side-effects of these drugs to the physicians who are treating neonates. The second step is to carry out empirical studies and multiple clinical trials to develop low-risk medicines for neonates, such as the production of suitable oral formulations for neonates (oral solutions instead of tablets) by pharmaceutical companies to reduce the prescription of unlicensed drugs. We also believe that a neonatal formulary containing all the information needed on the clinical use of neonatal drugs in Iran, (as present in some European countries like BNFc (*British* National Formulary for Children) in the UK), is necessary. The presence of a clinical pharmacist in the NICU of the hospital is necessary to confirm the low risk or the prescriptive use of the prescribed medication.

The limitation of the present study is that it was retrospective, which led to the withdrawal of a number of files of neonates admitted to the NICUs. In addition, since this study was only conducted in one province, the findings about the prescription of off-label and/or unlicensed drugs cannot be generalized to the whole country and further studies are needed. As the final point, because of the lack of information about the immediate and long-term complications of off-label and unlicensed medications, there is no reliable evidence available about possible harmful side effects of these drugs.

## Conclusion

The results of this study illustrate the similarities and differences in neonatal care protocols between Iran and other countries. Most of the neonates, especially preterm neonates, were exposed to off-label or unlicensed drugs. Unlicensed prescriptions were lower in our centers than in those investigated in other studies. So far, much effort has been exerted to reduce the inevitable prescription of these drugs. However, to identify the optimal and least risky treatments for the sensitive group of neonates, it is necessary for NICU medical teams, pharmaceutical companies, and other influential institutions to collaborate.

## Abbreviations

ADR: Adverse drug reactions
ATC: Anatomical therapeutic chemical
NICU: Neonatal Intensive Care Unit
RDS: Respiratory distress syndrome
TTN: Transient tachypnea of newborns
